# Thermal and Tribological Properties Enhancement of PVE Lubricant Modified with SiO_2_ and TiO_2_ Nanoparticles Additive

**DOI:** 10.3390/nano13010042

**Published:** 2022-12-22

**Authors:** Mohd Farid Ismail, Wan Hamzah Azmi, Rizalman Mamat, Hafiz Muhammad Ali

**Affiliations:** 1 Faculty of Mechanical and Manufacturing Engineering Technology, Universiti Teknikal Malaysia Melaka, Hang Tuah Jaya, Durian Tunggal 75150, Melaka, Malaysia; 2 Centre for Research in Advanced Fluid and Processes, Lebuhraya Tun Razak, Gambang, Kuantan 26300, Pahang, Malaysia; 3 Faculty of Mechanical and Automotive Engineering Technology, Universiti Malaysia Pahang, Pekan 26600, Pahang, Malaysia; 4 Mechanical Engineering Department, King Fahd University of Petroleum and Minerals, Dhahran 31261, Saudi Arabia

**Keywords:** nanolubricant, thermal conductivity, tribology, polyvinyl ether, refrigeration, nanofluid, compressor lubricant, PVE, SiO_2_, TiO_2_

## Abstract

The addition of nanoparticles may have a positive or negative impact on the thermal and tribological properties of base lubricant. The objective of this paper is to investigate the effect of nanoparticle dispersion in lubricant base in relation to its application in refrigeration system compressors. An investigation of tribological and thermal properties of nanolubricants for rolling piston rotary systems was carried out through four-ball tribology tests and thermal conductivity measurements. Nanolubricants dispersed with SiO_2_ and TiO_2_ nanoparticles were tested at various concentrations and temperatures. The changes in thermal conductivity and coefficient of friction (COF) were analyzed while wear weight loss was also calculated from wear scar size. A regression model of thermal conductivity enhancement was proposed for both types of nanoparticles. Zeta potential results show that nanolubricants have excellent stability. The thermal conductivity increases by the increment of nanoparticle concentration but decreases by temperature. The R-square for the regression model is more than 0.9952 with an average deviation not more than 0.29%. The COF for SiO_2_/PVE nanolubricant at 0.003 vol.% reduced 15% from the baseline. The COF for nanolubricants exceeds the result for base lubricants when the concentration is more than the threshold value. The optimum concentration of SiO_2_ and TiO_2_ nanoparticles improved the thermal and tribological properties of PVE lubricant and may offer an advantage when applied to refrigeration systems.

## 1. Introduction

Moving parts and interacting surfaces in mechanical motion may produce heat during the operation. Continuously scrubbing the surface may cause long-term harm to the component [1]. As a preventative measure, lubricants are introduced into the mechanical system to reduce friction and heat. The use of a lubricant in a mechanical system helps to save energy and prolong the component’s lifespan [2]. Therefore, lubricants are important to use for a variety of purposes, including cooling, sealing, and lubricating. In a vapor compression refrigeration (VCR) system, lubricant in the compressor works at high temperature and pressure. Typically, extreme pressure and anti-wear additives are used to enhance a fluid lubricant’s tribological performance by minimizing friction and surface degradation under harsh circumstances [3]. As most energies are consumed by the compressor, heat and tribological properties play an important role in determining the total energy consumption of the system.

The enhanced efficiency of machinery and equipment is one of the pillars of the global commitment to energy and environmental conservation. After the oil price shock of the 1970s, the demand for efficient and lower power-usage equipment rose as the price per unit of energy progressively increased [4]. As a result, the demand for high-efficiency appliances grew. Manufacturers, researchers, and other industrial players consistently work hard to fulfil the need of society for more environmentally friendly machines. The efficiency of an appliance can be improved through the introduction of high-efficiency components [5]. For instance, the efficiency of air conditioning can be improved by enhancing the operating components such as heat exchangers or compressors. However, changing component design is costly and time consuming. Therefore, nanoparticle dispersion technology was introduced to enhance system performance without replacing the existing components [6]. 

Nanoparticle dispersion technology is the method of dispersing less than 100 nm nanoparticles in a base fluid commercially known as a nanofluid [7]. The introduction of nanofluids to mechanical components has shown promising results for highly efficient systems [8]. The application of nanofluids in refrigeration systems was initiated by Wang et al. [9]. TiO_2_ nanoparticles were dispersed in a mineral oil base and the newly invented oil was named a nanolubricant. Then, the nanolubricant was applied to a R134a refrigerating system for performance measurement study. This experiment showed that the nanolubricant can improve the compatibility of lubricant with hydrofluorocarbon (HFC) refrigerant. Later, more studies were conducted related to the application of nanolubricant in refrigeration systems such as domestic refrigerators, heat pumps, air conditioning and chiller systems [10,11,12]. The nanolubricant studies also included the research in automotive air conditioning systems that was pioneered by Sharif et al. [13]. Base lubricants such as mineral oil (MO), polyol ester (POE), or polyethylene glycol (PAG) are commonly used for nanolubricant studies due to their solubility with the specific type of refrigerant. Meanwhile, the refrigerant is selected depending on its operating properties that fulfil the need of refrigerating system. Operating pressure, ambient temperature and type of compressor are amongst the primary factors that determine the selection of suitable refrigerant for a refrigeration system.

The initial research info nanolubricants focused on the physical properties of the fluid. The change of viscosity, thermal conductivity, and density of the lubricants was studied. Subsequently, tests for tribological properties were also undertaken in the research, as a response to the need to understand the friction effect of nanoparticles on the contact surfaces. The importance of the thermal conductivity property of nanofluids is undeniable since the pioneering research in nanoparticle dispersion technology [14,15]. In a refrigerant-nanolubricant mixture system, thermal conductivity always becomes one of the most important properties to be studied [16,17]. The presence of nanoparticles in the nanolubricant always positively impacts the performance of the VCR system as thermal conductivity values increase by the increment of nanoparticle concentration [18,19]. Some researchers combined the study on thermal conductivity with tribology [20,21,22]. As the tribological property increased the system’s ability to reduce friction in mechanical movement, thermal conductivity increases the system’s capacity to transfer heat efficiently [23]. Nanoparticle dispersion in lubricant has already been proven by some researchers as being able to increase the performance and reduce the energy consumption of a mechanical system [24]. Nanoparticles acted as a roller between two contact surfaces thus reducing the frictional effect. However, the volume concentration of nanoparticles must not exceed a threshold value. Values over this limit may cause tribological behavior to show lower improvement [25].

A relatively new compressor lubricant for the VCR system, polyvinyl ether (PVE), is considered to be a favorable alternative to POE oil. Unlike POE and PAG, the PVE oil does not hydrolyze water. Therefore, hydrolysis is not required in a system, especially useful in the VCR system. The filter dryer can be eliminated, and the long vacuuming process also can be shortened. Being a constituent of polymer base oil also brings advantages as the PVE retains its characteristics despite changes in viscosity [26]. However, studies on the properties of PVE are still lacking. Furthermore, nanolubricant research with PVE as lubricant base is far behind. Motozawa et al. [27] is the only report available of PVE nanolubricant with CuO as additive. The properties studied were thermal conductivity, viscosity, and dielectric constant. As far as the authors are aware, no report has been published to date on the tribological property of PVE nanolubricants. PVE’s role as a lubricant was previously intended as compressor lubricant in air conditioning systems. In such applications, tribological, thermal, and rheological properties of lubricant play an important role in determining the performance of such a system. However, the pure PVE lubricant is reasonably low in thermal conductivity and viscosity [28]. Nanoparticle additives solve this issue by increasing the thermal conductivity as well as improving the tribological property of lubricant [29,30]. SiO_2_ and TiO_2_ nanoparticles were amongst the best nanoparticle candidates able to improve the performance of VCR system [31,32]. In this study, the thermal and tribological properties of PVE nanolubricant will be investigated. The SiO_2_ and TiO_2_ nanoparticles will be used as an additive to discover the improvement in thermal and friction properties.

## 2. Experimental Methods

### 2.1. Nanolubricant Preparation and Stability Evaluation

The PVE lubricant used in this paper was procured from Idemitsu Kosan Co., Ltd. This lubricant is commonly used for HFC refrigeration compressors, especially for residential air conditioning systems. Table 1 summarizes the chemical-physical properties of PVE lubricant. The 99.9% purity SiO_2_ nanoparticle used in this paper was obtained from HWNANO (Hongwu International Group Ltd., Guangzhou, China), while the TiO_2_ nanoparticle was from DKNANO (Beijing Deke Daojin Science and Technology Co., Ltd., Beijing, China) and also has the same purity percentage. Table 2 summarizes the properties of nanoparticles used in this paper. The SiO_2_ and TiO_2_ nanolubricant in this paper were prepared using a two-step method. The measurement of nanoparticles for different volume concentrations is calculated using Equation (1)

**Figure 1:**
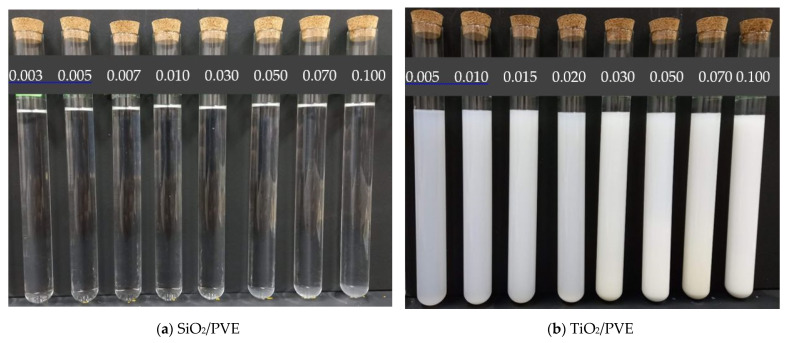
Nanolubricants after preparation at different volume concentration (%).

(1) ϕ=mnρnmnρn+mlρl×100%

where ϕ is the concentration in volume percent, $m_{l}$ and $m_{n}$ are the masses of PVE lubricant and nanoparticle, respectively, while $\rho_{l}$ and $\rho_{n}$ are the density of PVE lubricant and nanoparticle, respectively. Nanoparticles were weighed using a high precision (±0.0001) weighing scale. The minimum volume of each concentration prepared was 100 mL. The nanoparticles were poured into stirred lubricant very gradually to avoid agglomeration. The stirring process took half an hour before the nanolubricants were transferred to the homogenizing process [33]. The homogenizer equipment used in this study was an ultrasonic water bath (Fisherbrand FB15051) with 200W power and 50 kHz constant frequency. The homogenizing process took 1 h for SiO_2_ and 5 h for TiO_2_ nanolubricants. Figure 1 shows part of the prepared sample of both types of nanolubricants. The photos for visual stability evaluation were captured just after nanolubricant preparation was completed. The stability of nanolubricants was also measured by using the zeta potential method as suggested by [34]. 5 mL of sample from a 0.01% concentration of both type nanolubricants were separated from the main beaker for this purpose. As the concentration is not a factor that determines the zeta potential measurement, only one concentration was tested that represents the stability of all nanolubricants. The zeta potential measurement was repeated three times for each sample and the average value was calculated and is presented. To characterize and identify of nanolubricants, a small amount of the samples was sent to the laboratory for microscopy evaluation. A TECNAI G2 F20 X-Twin high-resolution transmission electron microscope (HRTEM) was used in this study to ensure the presentation, size, shape, and position of the nanoparticles in the prepared sample.

The SiO_2_/PVE nanolubricants were prepared at concentrations from 0.003% to 0.1% while TiO_2_/PVE nanolubricants were from 0.005% to 0.1%. For thermal property measurement, the minimum concentration was conducted at 0.01% for both types of nanolubricants. The limited measurement capability of thermal conductivity devices does not allow lower concentrations to be tested as the deviation of thermal conductivity is too small compared to the measurement error. For tribological measurement, the tests were conducted starting from the 0.003% and 0.005% concentrations for SiO_2_/PVE and TiO_2_/PVE nanolubricant, respectively.

### 2.2. Thermal Conductivity Measurement

The thermal conductivity measurement was conducted using TCi Thermal Conductivity Analyzer by C-Therm. It has less than 5% accuracy for the measurement range between 0 to 500 W/mK. The samples were measured between 30 to 80 °C. A 35 mL sample was filled in a sample cell. The thermal sensor was connected and located properly in the dedicated oven. The heat imposed on the sample and thermal effusivity was measured. The thermal conductivity was calculated from the effusivity measurement together with heat capacity and density as input. The device recorded one thermal conductivity value for every 0.2 °C increment. This method used in this thermal conductivity measurement applied Modified Transient Plane Source (MTPS) that conforms to ASTM D7984. The measurement for each nanolubricant was repeated three times to confirm the repeatability of measurement, and the average value was taken as final experimental data. The thermal conductivity models by Hamilton and Crosser [37], Redhwan et al. [18], and Zawawi et al. [19] shown in Table 3 are used to verify the thermal conductivity of SiO_2_-TiO_2_/PVE nanolubricants.

### 2.3. Tribology Measurement

**Figure 2:**
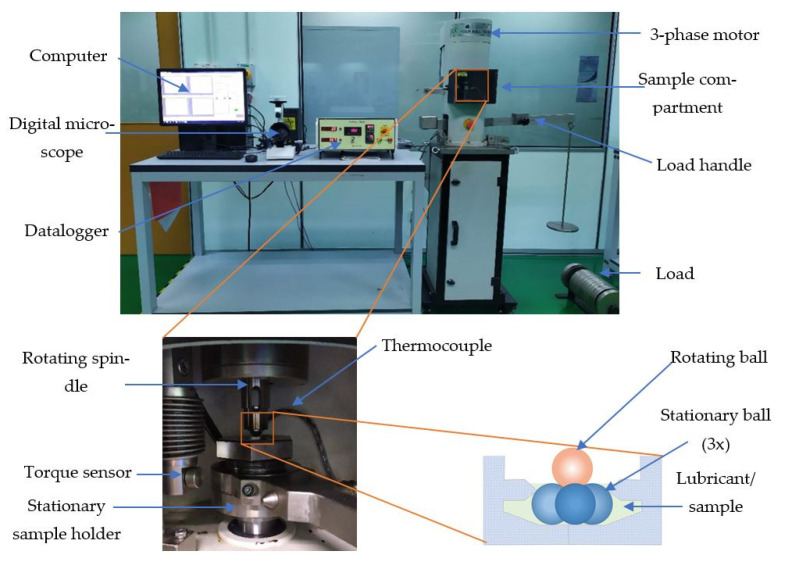
Four-ball Tribology Testing Machine.

The tribological properties investigation was carried out using the Koehler Four-Ball Tribo Tester for PVE base lubricant and nanolubricant with SiO_2_ and TiO_2_ nanoparticles additive. This method was also extensively used by Sanukrishna et al. [23], Kamel et al. [3], and Wang and Wang [38] for nanolubricant studies with different lubricant bases and additives. The relative investigation was carried out according to ASTM D4172. The steel balls are chromium steel ball G20 that are manufactured in accordance with the ISO 3290 protocol. Hexane was used to clean the balls and tools from any oil or debris. Three balls were located inside the oil handler and tightened. The lubricant submerged the balls and the contact surface touched the fourth ball from the top. A load of 40 Nm was applied to the balls while the rotating speed was set at 1200 rpm. A thermocouple was connected to the oil handler to measure the lubricant temperature. A heater with an automatic temperature controller was used to maintain the lubricant temperature at 75 °C. The equipment and set up for the tribological testing conducted in the current study is presented in Figure 2.

Wear scar morphology is one of the methods that can be used to evaluate the anti-wear characteristics of lubricants. The wear scar diameter (WSD) provides information about friction load capacity and the severity of wear due to friction. Greater wear rate can be determined by the bigger size of WSD and vice versa. The wear weight loss ($W_{loss}$) can be determined using Equations (2) to (4)

(2) $$W_{loss}=W_{int}-W_{final}$$

(3) $$W_{loss}=\frac{1}{3}\pi h_{l}^{2}\left(\frac{3}{2}D-h_{l}\right)$$

(4) $$h_{l}=\frac{D}{2}-\sqrt{{\left(\frac{D}{2}\right)}^{2}-{\left(\frac{d}{2}\right)}^{2}}$$

where $W_{int}$ in grams (g) is the initial weight of the ball before the test; $W_{final}$ in grams (g) is the final ball weight after the test; D is the diameter of the ball which is 12.7 mm; d is the diameter of wear scar in mm and $h_{l}$ is the height of loss volume. The weight of each ball was measured using a high-accuracy weighing scale and recorded for volume loss calculation later.

### 2.4. Uncertainty Analysis

The uncertainties for the thermal conductivity analyzer and four-ball tribology tester are presented in Table 4. The uncertainties of measurement are analyzed with less than 1.09%. 

## 3. Results and Discussion

### 3.1. Stability of Nanolubricants

**Figure 3:**
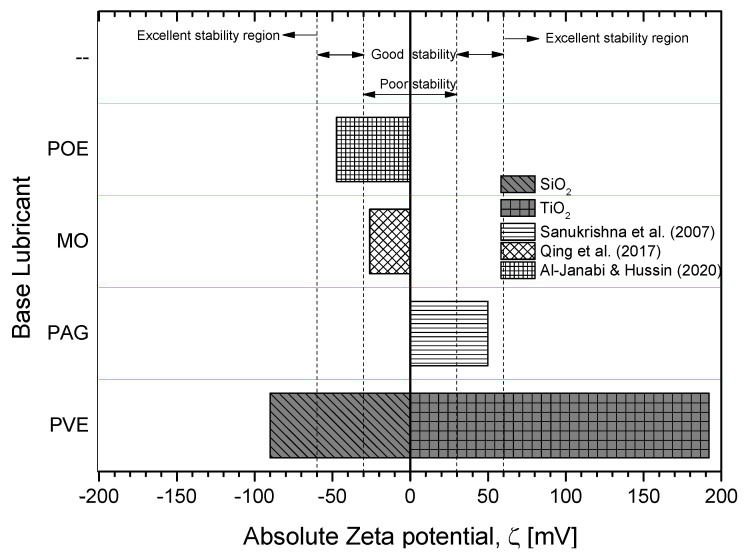
Zeta Potential measurement comparison of present study nanolubricant with literature [39,40,41].

Visual observation of the prepared nanolubricants showed that the SiO_2_/PVE and TiO_2_/PVE nanolubricants were stable for up to 30 days. No separation occurred between lubricant and nanoparticles. However, a small amount of sedimentation was observed at the bottom of the nanolubricant showing that there was some gravitational effect on the dispersed nanoparticles. The initial murky white nanolubricants became clearer 30 days after the preparation. The zeta potential test on one sample of each type of nanolubricant was conducted on the same day as preparation. The results are shown in Figure 3 [39,40,41]. SiO_2_/PVE nanolubricant returned a zeta potential value of −90 mV while TiO_2_/PVE was 192 mV. According to Ghadimi et al. [42], a zeta potential of more than 60 mV is considered to have excellent stability. The same figure also compares to the zeta potential value from literature with different lubricant bases [39,40,41]. Based on the visual observation and zeta potential test on the nanolubricants, both types of nanolubricants have excellent stability up to 30 days.

**Figure 4:**
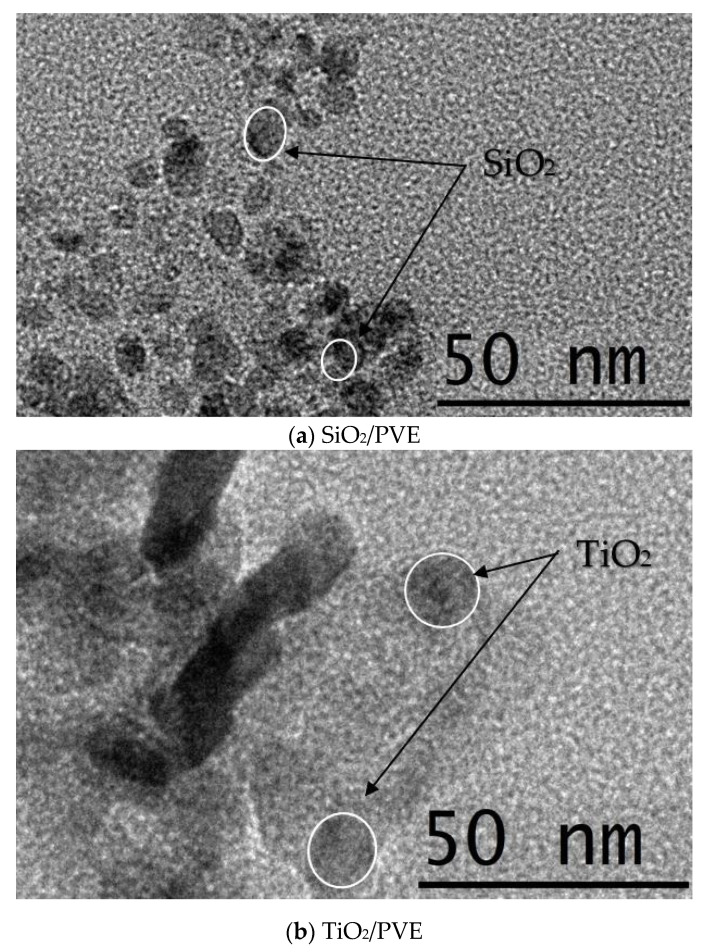
TEM image of nanolubricants.

Figure 4 shows the results of the HRTEM image captured from the prepared nanolubricants. The image was captured with a resolution of ×88,000 so that the size of the nanoparticle dispersed in PVE lubricant was able to be determined. The image confirmed the average size of SiO_2_ nanoparticles as 15 nm while the TiO_2_ was 30 nm. The nanoparticles in the lubricant base were uniformly distributed with some agglomeration present.

### 3.2. Thermal Conductivity of Nanolubricants

**Figure 5:**
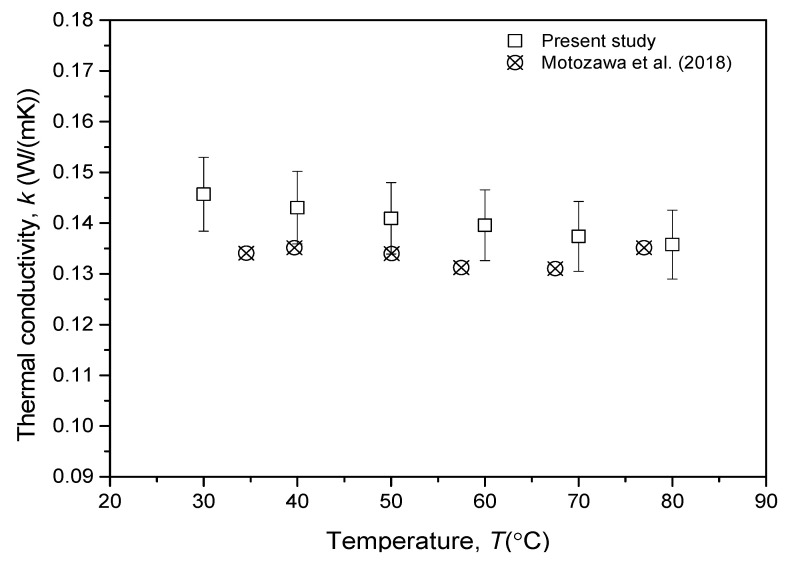
Thermal conductivity verification of PVE lubricant with available references [27].

Figure 5 depicts the comparison of thermal conductivity measurement of pure PVE lubricant in the present study with available references from the literature. The comparison was made as a verification of the current thermal conductivity measurement with the report from previous findings by Motozawa et al. [27]. Even though the PVE lubricant used in this current study was not the same as their PVE lubricant, in that they were of different manufacturers, both lubricants were of the same grade. The comparison was made at environment temperatures between 30 and 80 °C. The results showed that the present thermal conductivity measurements were within a 5% deviation from the measurement made by Motozawa et al. [27]. The results also revealed that the thermal conductivity of pure PVE lubricant was slightly decreased by the increment of temperature.

**Figure 6:**
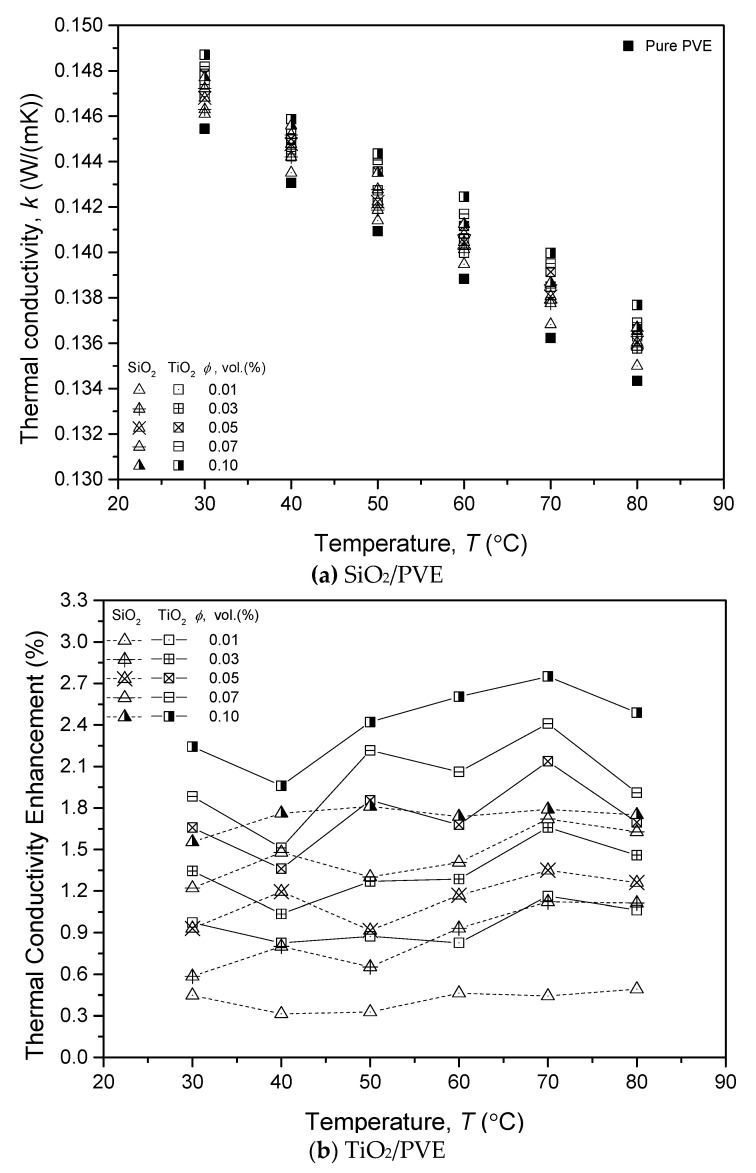
Thermal conductivity (**a**) measurement (**b**) increment for SiO_2_/PVE and TiO_2_/PVE nanolubricants.

The thermal conductivity measurement of SiO_2_ and TiO_2_ nanolubricant also shows a similar trend to pure PVE lubricant. Figure 6a,b present the thermal conductivity measurement and its enhancement for both types of nanolubricants, respectively. The thermal conductivity of pure PVE was also plotted as a comparison to the measurement for nanolubricants. The results indicated that nanolubricants had higher thermal conductivity than pure PVE lubricants at all measured temperatures. However, the value of thermal conductivity decreased when the temperature increased. At 30 °C, the thermal conductivity was the highest. The results also revealed that TiO_2_/PVE nanolubricants always had higher thermal conductivity than that of SiO_2_/PVE at the same concentration. The TiO_2_ nanoparticles had higher thermal conductivity (8.4 W/m·K) values compared to SiO_2_, which was only 4 W/m K as presented in Table 2. The thermal conductivity measurement of SiO_2_ and TiO_2_ nanolubricant also showed a similar trend for pure PVE lubricant.

The thermal conductivity of nanolubricants at all measured concentrations was always higher than that of pure PVE lubricant, as depicted in Figure 6b. The enhancement trend shows that the increment of nanoparticle concentration may increase the thermal conductivity. It was also discovered that temperature did not influence the amount of thermal conductivity increment of the nanolubricants. There was no significant trend of thermal conductivity changes when the temperature changed. The current finding was aligned with thermal conductivity enhancement reported by Sharif et al. [13] who studied the properties of PAG lubricant dispersed with Al_2_O_3_ nanoparticle. The thermal conductivity enhancement of PVE nanolubricants was considered as insignificant as the maximum increment was less than 3%, with a volume concentration of 0.10% TiO_2_ nanoparticles. The presence of nanoparticles in PVE lubricant influenced the heat-conducting behavior as the nanoparticles themselves had a better capability of conducting heat. However, when the heat was applied to the liquid, the nanoparticles and lubricant molecules started to vibrate and move apart from each other. Therefore, the thermal conductivity between nanoparticles and molecules decreased as the possibility of particle collision decreased at a higher temperature.

### 3.3. Thermal Conductivity Regression Model Analysis

**Figure 7:**
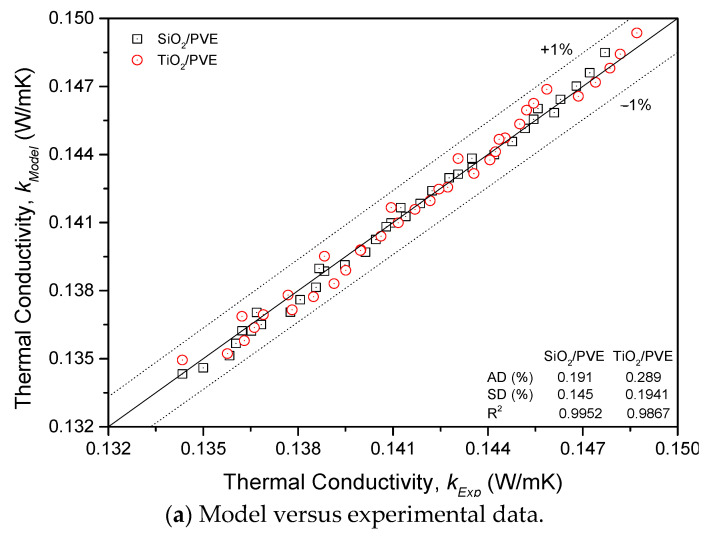

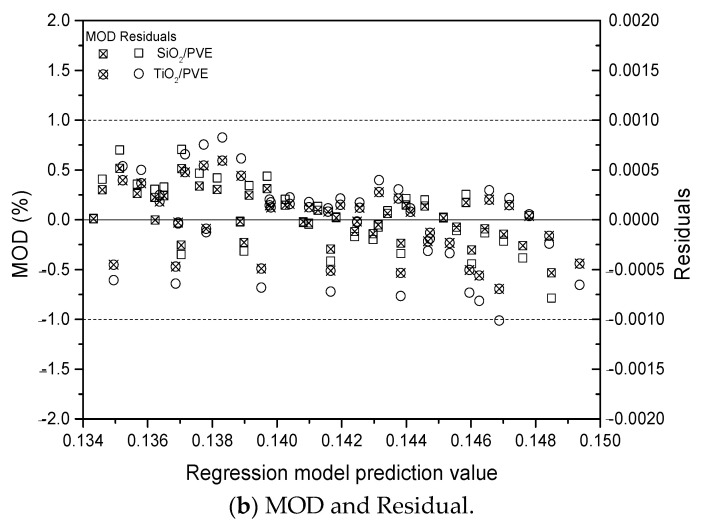
Regression model and statistical analysis of nanolubricants thermal conductivity.

From the experimental measurement results, regression analysis was performed on the thermal conductivity ratio of nanolubricant to pure PVE lubricant. Two effective thermal conductivity equations were proposed for SiO_2_/PVE and TiO_2_/PVE nanolubricant as presented in Equations (5) and (6), respectively. Both equations are valid for nanoparticle concentrations of 0≤ϕ≤0.1% and temperatures between 30≤T≤80 ℃. Figure 7a shows the plot determining thermal conductivity using the equation model ($k_{Model}$) versus the experiment data ($k_{Exp}$). There was a good agreement of the regression equation with the experimental data where the deviation between those thermal conductivity values were ± 1%. The regression was convincing, with an R^2^ value for both nanolubricants of more than 0.9867. The AD was not more than 0.3%, and the SD was less than 0.2%.

(5) $$k_{r}=\frac{k_{nl}}{k_{l}}=\frac{{\left(1+\frac{\phi }{100\%}\right)}^{20}}{{\left(0.1+\frac{T}{80\;℃}\right)}^{0.001}}$$

(6) $$k_{r}=\frac{k_{nl}}{k_{l}}=\frac{{\left(1+\frac{\phi }{100\%}\right)}^{21}}{{\left(0.1+1.1\frac{T}{80\;℃}\right)}^{0.003}}$$

Further investigation of the regression model was conducted using the statistical method as presented in Figure 7b. The margin of deviation (MOD) of each point was calculated using Equation (7) and plotted to illustrate the size of the deviation percentage. In addition, the difference between the experimental data and the predicted model was also presented as residuals in the same figure. The distribution of MOD and residuals of nanolubricants did not exceed ± 1% and 0.001, respectively. The distribution of points was random without any significant trend. The ANOVA was also conducted for SiO_2_/PVE and TiO_2_/PVE nanolubricants regression model and presented in Table 5. It was shown that the regression model was very accurate based on the statistical analysis performed on it.

(7) $$MOD=\;\left[\frac{{\left(k_{nl}\right)}_{Exp}-{\left(k_{nl}\right)}_{Model}}{{\left(k_{nl}\right)}_{Exp}}\right]\times 100\%$$

**Figure 8:**
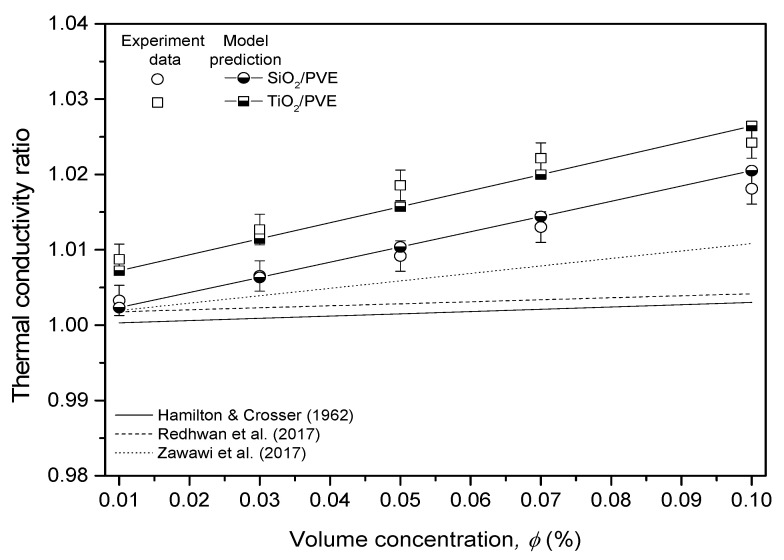
Comparison of thermal conductivity with literature and predicted model at 50 °C [18,19,37].

To ensure that the current experimental data and the regression model were correct, a comparison was made with the previously published data, as shown in Figure 8. The model suggested by Hamilton and Crosser [37] was used as the main reference in this comparison. In addition, the regression model using PAG as the base lubricant was also used as a reference, as their model was valid for the concentration studied in this research [18,19]. For a fair comparison, the temperature for all data was fixed at 50 °C. The model and experiment data in the current study showed a significant trend with the literature, where the thermal conductivity increases with the increment of volume concentration. The thermal conductivity for nanolubricants was always higher than that of pure lubricants showing the significant impact of nanoparticle dispersion in the modified lubricant. The model proposed for both SiO_2_/PVE and TiO_2_/PVE nanolubricants had good agreement with the experimental data with an error of less than 0.2%.

### 3.4. Frictional Properties

The frictional property of pure PVE and its nanolubricants was evaluated by the coefficient of friction changes. The COF was calculated from the frictional torque ($T_{f}$) in kg·cm and the normal load ($W_{N}$) in kg applied to the balls expressed in Equation (8). The equation was obtained from the present experimental data.

(8) $$COF=2.23004\frac{T_{f}}{W_{N}}$$

**Figure 9:**
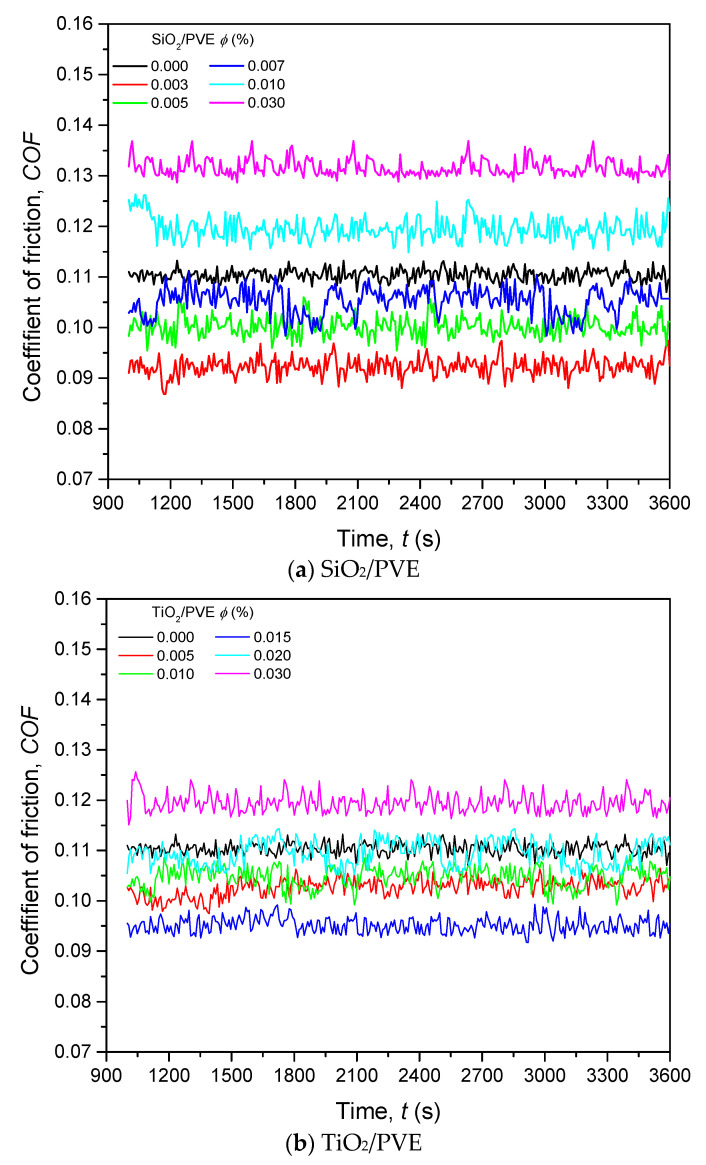
Coefficient of friction for nanolubricants in comparison to pure PVE lubricant.

Figure 9a,b illustrates the COF for SiO_2_/PVE and TiO_2_/PVE nanolubricants with different concentrations, respectively. The COF of pure PVE is also presented as 0.000 concentration in comparison to nanolubricants.

Even though the frictional test was conducted for a complete hour, the data are presented only from 1000 to 3600 s. During this period, the COF showed a consistent reading. In Figure 9a, the COF for SiO_2_/PVE nanolubricants with the concentration of 0.003%, 0.005%, and 0.007% show lower values compared to the COF of pure PVE. Other samples showed higher COF. This trend indicates that nanolubricants below 0.007% concentration had better COF than that of the pure lubricant. A similar trend happened for TiO_2_/PVE nanolubricants in Figure 9b with a small difference. The only TiO_2_/PVE nanolubricant that had higher COF than that of pure lubricant was the one with a 0.03% concentration. Because of this, the tribological measurement for nanolubricants was stopped at 0.03% without further continuation. It was expected that the COF of more than this limit would become higher and would not contribute any significant results to the current study.

**Figure 10:**
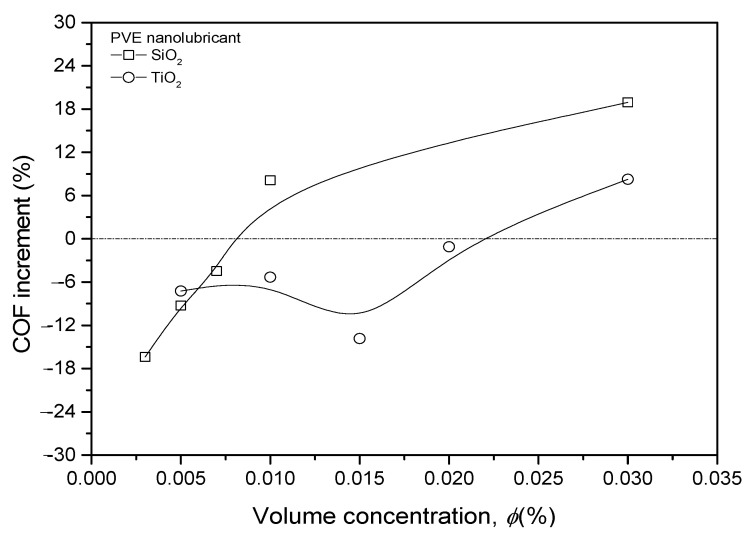
Nanolubricants coefficient of friction changes compared to PVE lubricant.

For a better understanding of the COF changes, the difference between nanolubricants and pure PVE was calculated and is presented in Figure 10. The COF of nanolubricants was normalized with the COF of pure PVE lubricant for all prepared samples. The COF of SiO_2_/PVE nanolubricants shows an increment trend from 0.003% to 0.03% concentration. A linear trend is observed for the first four points before the gradient decreases until it reaches 18% higher than that for pure PVE for 0.03% concentration. The COF changes also illustrate that the SiO_2_/PVE nanolubricant with low concentration (less than 0.007%) shows a decrement compared to that of the base lubricant. The COF trend of TiO_2_/PVE has a slightly different trend. The first four concentrations show a decrement compared to that of base lubricant. However, the COF value is not linear. The nanolubricant with a concentration of 0.015% had the lowest COF. The linear COF increment appears from this point until it crosses the baseline at 0.03%.

The COF of nanolubricants was expected to decrease at low concentrations due to the small amount of nanoparticles present in the lubricant. The inclusion of a small amount of nanoparticles in nanolubricant may fill the gap produced by the wear effect between solid surfaces. Nanoparticles may create rolling and mending effects between surfaces together with the lubricant. However, there was a threshold for the usefulness of nanoparticles inside the lubricant. Exceeding this limit may result in the nanoparticle becoming abrasive against the contact surface. This has happened at a high concentration, i.e., 0.03%.

### 3.5. Wear Scar Analysis

**Figure 11:**
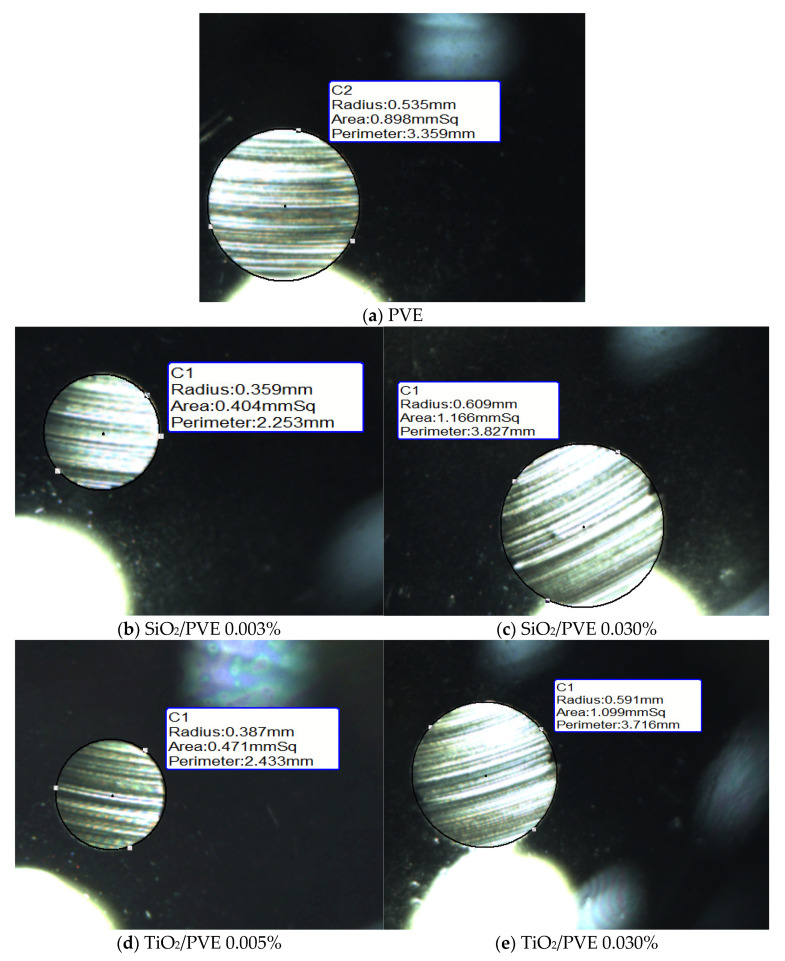
Regression model and statistical analysis of nanolubricants thermal conductivity.

The wear scar developed on the ball surfaces due to the rotational motion of the ball in the tribological experiment. A round scar formed on all three bottom balls, while a ring scar developed on the surface of the top ball. The size of WSD depends on the anti-wear capability of the lubricant as the surfaces were in contact. Smaller WSD indicate better lubrication performance due to the lower frictional effect acting on the ball surfaces. The WSD for each sample was inspected and measured just after completing the frictional test. The measurement recorded the radius, the area, and the perimeter of the wear scar of each stationary ball. The top ball had a circular line on its surface that was in contact with the three bottom balls. Figure 11 represents pictures captured of several wear scars on the surface of the balls.

Since the operating time for each lubricant was constant (3600 s), the size of the wear scar was solely affected by the anti-wear characteristics of the lubricant sample. A bigger WSD means that the lubricant sample has poor anti-wear characteristics and vice versa. The WSD for SiO_2_/PVE 0.003% has a smaller size than the 0.030% concentration. The same result goes for TiO_2_/PVE nanolubricant with a concentration of 0.005% compared to 0.030%. The WSD for pure PVE was between these two concentrations. This WSD results in agreement with COF results in Figure 9. Nanolubricants with lower concentrations showed smaller WSD compared to those for higher concentrations. 

**Figure 12:**
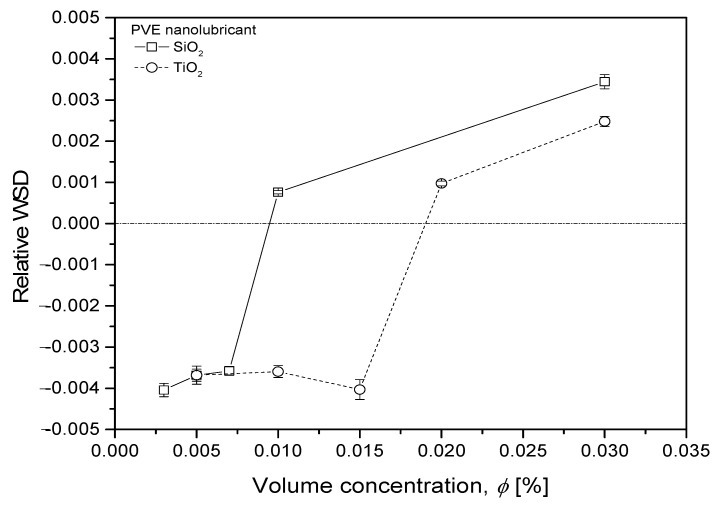
WSD changes relative to pure PVE lubricant for nanolubricants.

The full results of WSD are presented in Figure 12. For comparison purposes, the WSD for nanolubricants were compared relative to WSD for pure PVE lubricant. The data in this figure show that wear scar sizes for nanolubricants with concentrations less than 0.010% and 0.020% for SiO_2_/PVE and TiO_2_/PVE, respectively, were smaller than the wear scar size for the base lubricant. Even the scar size became bigger when the concentration increased. For SiO_2_/PVE nanolubricants, the sample with a concentration of 0.003% was the smallest, while for TiO_2_/PVE, 0.015% was the smallest. The WSD trends were almost similar to the COF changes presented in Figure 10. The wear scar size supports the findings of COF, where the SiO_2_ and TiO_2_ nanoparticles behaved as anti-wear agents in lubricants at low volume concentrations. By the increment of concentration, the abrasive behavior of nanoparticles increased until the abrasive behavior had a a higher impact than the anti-wear behavior, thus producing more disadvantages to the overall process. Because of this, it was suggested that the tribological measurements stop at concentrations of 0.030%.

## 4. Conclusions

The present paper focuses on the effect of thermal and tribological properties by nanoparticle dispersion in lubricant. SiO_2_ and TiO_2_ nanoparticles were dispersed homogenously in PVE lubricant. Nanolubricants were characterized and their stability was evaluated. Thermal properties were investigated from 30 to 80 °C while tribological properties were studied employing the four-ball method. The thermal conductivity increases by the increment of nanoparticles concentration but decreases with temperature increment for both SiO_2_/PVE and TiO_2_/PVE nanolubricants. Very promising results were obtained from nanolubricants with the concentration of 0.005 vol.% and 0.015 vol.% for SiO_2_/PVE and TiO_2_/PVE respectively, with a significant COF reduction of around 15%. The friction reduction was the effect of contact area reduction by the nanoparticle’s morphology acting on the surface of two mechanical components that were in contact. The study also identifies the threshold value of nanoparticles as an additive for SiO_2_ and TiO_2_; 0.010 vol.% and 0.020 vol.%, respectively; to exceed this may cause an increment in friction. The wear scar radius on the ball surface confirms the friction reduction effect by nanoparticles. The thermal conductivity regression model was also proposed for both types of nanolubricants with a high confidence level. SiO_2_ and TiO_2_ additives in nanolubricant improved the thermal and tribological properties of PVE oil with the condition that the nanoparticle concentration does not exceed the threshold value.

## Figures and Tables

**Table 1:** Properties of base lubricant: [26].

Property	PVE
Dynamic Viscosity, mm^2^/s @ 40 °C	68.1
Dynamic Viscosity, mm^2^/s @ 100 °C	8.04
Viscosity index	84
Pour Point, °C	−37.5
Flash point, °C	204
Density, kg/m^3^ @ 15 °C	936.9

**Table 2:** Properties of nanoparticles [35,36].

Property	Nanoparticles	
	SiO_2_	TiO_2_
Density, ρ [kg/m^3^]	2220	4230
Thermal conductivity, *k* [W/mK]	4	8.4
Molecular mass, *M* [g/mol]	60.08	79.87
Average diameter, $d_{p}$ [nm]	15	30–50
Specific surface area [m^2^/g]	250	-
Specific heat, *c_p_* [J/kgK]	745	692

**Table 3:** Thermal conductivity model.

Models	Correlations
Hamilton and Crosser [37]	$k_{r}=\frac{k_{eff}}{k_{bf}}=\frac{k_{p}\;+\;\left(n-1\right)k_{bf}\;-\;\left(n-1\right)\varphi \left(k_{bf}\;+\;k_{p}\right)}{k_{p}\;+\;\left(n-1\right)k_{bf}\;+\;\varphi \left(k_{bf}\;+\;k_{p}\right)}$
Redhwan et al. [18]	$k_{r}=\frac{k_{eff}}{k_{bf}}=1.2{\left(1+\frac{\phi }{100}\right)}^{0.04}{\left(1+\frac{T-273}{80}\right)}^{-0.01}$
Zawawi et al. [19]	$k_{r}=\frac{k_{eff}}{k_{bf}}={\left(1+\frac{\phi }{100}\right)}^{9.8}{\left(1+\frac{T-273}{80}\right)}^{0.002}$

**Table 4:** The summary for the uncertainty.

Parameters	Accuracy	Values Measured		Uncertainty (%)
		Min	Max	
Thermal conductivity, *k* [W/mK]	±0.001	0.136	0.148	0.67–0.73
Coefficient of friction, *COF*	±0.001	0.091	0.139	0.72–1.09

**Table 5:** Analysis of variance (ANOVA) for thermal conductivity of proposed regression.

	Nanolubricant	df	SS	MS	F	Significance F
Regression	SiO_2_/PVE	1	0.0005762	0.00058	7059.1402	5.07 × 10^−41^
	TiO_2_/PVE	1	0.000584	0.000584	2520.0464	1.77 × 10^−33^
Residual	SiO_2_/PVE	34	2.775 × 10^−6^	8.2 × 10^−8^		
	TiO_2_/PVE	34	7.879 × 10^−6^	2.32 × 10^−7^		
Total	SiO_2_/PVE	35	0.0005789			
	TiO_2_/PVE	35	0.0005918			

## Data Availability

Not applicable.
